# Micro computed tomography with and without contrast enhancement for the characterization of microcarriers in dry and wet state

**DOI:** 10.1038/s41598-021-81998-8

**Published:** 2021-02-02

**Authors:** Sébastien de Bournonville, Liesbet Geris, Greet Kerckhofs

**Affiliations:** 1 Division of Skeletal Tissue Engineering, KU Leuven, Leuven, Belgium; 2 Biomechanics Section, Department of Mechanical Engineering, KU Leuven, Leuven, Belgium; 3 Biomechanics Research Unit, ULiège, Liège, Belgium; 4 Biomechanics Lab, Institute of Mechanics, Materials and Civil Engineering, UCLouvain, Louvain-la-Neuve, Belgium; 5 Department Materials Engineering, KU Leuven, Leuven, Belgium; 6 Institute of Experimental and Clinical Research, UCLouvain, Woluwé-Saint-Lambert, Belgium

**Keywords:** Biomedical engineering, Biomaterials, Tissue engineering, Materials science

## Abstract

In the field of regenerative medicine, microcarriers are used as support matrix for the growth of adherent cells. They are increasingly recognised as promising biomaterials for large scale, cost-effective cell expansion bioreactor processes. However, their individual morphologies can be highly heterogeneous which increases bioprocesses’ variability. Additionally, only limited information is available on the microcarriers’ 3D morphology and how it affects cell proliferation. Most imaging modalities do not provide sufficient 3D information or have a too limited field of view to appropriately study the 3D morphology. While microfocus X-ray computed tomography (microCT) could be appropriate, many microcarriers are hydrated before in-vitro use. This wet state makes them swell, changing considerably their morphology and making them indistinguishable from the culture solution in regular microCT images due to their physical density close to water. The use of contrast-enhanced microCT (CE-CT) has been recently reported for 3D imaging of soft materials. In this study, we selected a range of commercially available microcarrier types and used a combination of microCT and CE-CT for full 3D morphological characterization of large numbers of microcarriers, both in their dry and wet state. With in-house developed image processing and analysis tools, morphometrics of individual microcarriers were collected. Also, the morphology in wet state was assessed and related to accessible attachment surface area as a function of cell size. The morphological information on all microcarriers was collected in a publicly available database. This work provides a quantitative basis for optimization and modelling of microcarrier based cell expansion processes.

## Introduction

As the number of clinical trials for cell-based therapies is increasing, so does the need for robust and cost-effective large-scale biomanufacturing strategies. From 2014 to 2018, the number of clinical trials initiated grew by 32%^1^ and as of June 2019, more than 1000 clinical trials are underway, amongst which more than 600 for cell and gene-modified cell therapies^2^. Most of these therapies require the robust production of large number of cells, up to several billions, especially in the case of allogeneic therapies^3,4^. To overcome this challenge, an increasing number of innovative bioreactors are being developed, with more process monitoring and control features^5^. Over the last years, the use of microcarriers for the large scale expansion of adherent cells has been increasingly reported^6^. The main advantage of such culture methods is the combination of a high ‘attachment surface area to culture volume’ ratio with the homogeneity of suspension cultures^7^. This makes it a promising solution for scalable and cost-efficient large scale biomanufacturing of adherent cells^8–11^. Table 1 gathers most of the commercially available microcarriers, reported for cell expansion bioprocesses. However, challenges remain to make the use of this technology an industrial reality.

**Table 1:** Commonly used commercially available microcarriers for cell expansion processes.

Manufacturer & Microcarrier	Number of microcarriers/g dry weight	Density (g/ml) *	Particle size (µm)*	Surface area (cm^2^/g dry weight) *	Additional information on morphology and matrix composition	Reported use
*Microcarriers included in this study*						
GE Healthcare – Cytodex 1	4.3*10^6^	1.03	190	4400	Non-porous, swelling spherical beads. Cross-linked dextran and diethylaminoethyl (DEAE) matrix	^16,28–32^
GE Healthcare – Cytodex 3	3.0*10^6^	1.04	175	2700	Non-porous, swelling spherical beads. Dextran matrix	^29,32–36^
Percell Biolytica – CultiSpher S	8.0*10^5^	1.03 †	255 †	7500	Macro-porous, swelling beads. Gelatine matrix	^31,32,36–41^
Corning – Dissolvable Synthemax II	n.a	1.025 †	250 †	5000	Non-porous swelling beads. Polygalacturonic acid (PGA) polymer chains cross-linked via calcium ions and coated with Synthemax II. Also available untreated or with Collagen coating	^14^
Corning – Polystyrene untreated	n.a	1.026	168.5 †	360	Non-porous, spherical beads. Polystyrene matrix. Also available with different coatings (Collagen, positively charged, CellBind, Synthemax II)	^10,42,43^
Pall SoloHill – Collagen	4.6*10^5^	1.026 †	168.5 †	360	Non-porous, spherical beads. Cross-linked polystyrene matrix coated with collagen type 1	^32,36,44,45^
Pall SoloHill – FACT 3	4.6*10^5^	1.026 †	168.5 †	360	Non-porous, spherical beads. Cross-linked polystyrene matrix coated with collagen type 1 and proprietary surface modification	^32,44^
Pall SoloHill – Hillex 2	n.a	1.120 †	180 †	515	Non-porous, spherical beads. Polystyrene matrix modified with cationic amine	^32,44,45^
Pall SoloHill – Plastic	4.6*10^5^	1.026 †	168.5 †	360	Non-porous, spherical beads. Styrene co-polymer matrix	^32,45–48^
Pall SoloHill – Plastic Plus	4.6*10^5^	1.026 †	168.5 †	360	Non-porous, spherical beads. Styrene co-polymer matrix with electric charge	^14,32,45,49^
Pall SoloHill – Star Plus	4.6*10^5^	1.026 †	168.5 †	360	Non-porous, spherical beads. Cross-linked polystyrene matrix	^45^
*Microcarriers not included in this study*						
GE Healthcare – Cytopore 2	3.0*10^6^	1.03	230	11,000	Macro-porous, swelling non-spherical beads. Cellulose and DEAE matrix	^31,32^
Pall SoloHill – ProNectin F	n.a	n.a	120 †	480	Non-porous, spherical beads. Polystyrene coated with recombinant RGD-containing protein	^32,50^
Pall SoloHill – Glass	n.a	n.a	168.5 †	360	Non-porous, spherical beads. Cross-linked polystyrene coated with High silica glass	
MP Biomedical – RapidCell	n.a	1.03 †	180 †	325	Non-porous, spherical beads. Glass matrix	^51^
Tosoh Biosciences – Tosoh 65 PR	n.a	n.a	65	4200	Non-porous, spherical beads. Hydroxylated methacrylate with Tresyl ligand derivatized and Protamine sulphate surface treatment	^30^
Tosoh Biosciences – Tosoh 10 PR	n.a	n.a	10	9000	Non-porous, spherical beads. Hydroxylated methacrylate with Tresyl ligand derivatized and Protamine sulphate surface treatment	^30^
Nunc – Biosilon	n.a	1.05	190	225	Non-porous, spherical beads. Polystyrene matrix	^16^
Whatman – DE-53	n.a	1.1	L: 130, D: 35	6800	Non-porous cylindrical microcarriers. Cellulose matrix with DEAE surface treatment. Also available with lower charge density (DE-52) or different surface treatments (QA-52, CM52)	^30^
New Brunswick – Fibra-Cel	n.a	n.a	6000	120	Macro-porous, disk-shape microcarriers. Polyester non-woven fibre and polypropylene matrix	^31^

*Metrics in fully hydrated state (where applicable). †Average calculated from the range provided by the manufacturer. n.a.: Not available.

Harvesting cells from the microcarriers with a high efficiency while maintaining their quality, is one of them, hence making it one of the most critical steps of the process^12^. Nowadays, new types of dissolvable and external stimulus-responsive microcarriers (i.e. thermo-, electro- or light-responsive) are being developed in the hope of overcoming this limitation^13–21^. An additional challenge is the high morphological heterogeneity of most microcarriers within one production batch, and, more importantly, the lack of quantitative information on their morphological properties, despite being critical process parameters^22–24^. The heterogeneity introduces external variability in the system and hampers stochastic analyses of bioprocesses focused on cell-related variability. Additionally, it prevents the study and optimization of cell attachment behaviour on new substrate morphologies.

Furthermore, many of the microcarriers developed in the field need to be hydrated in a preparation solution in order to swell before being used in process (cf. Table 1). This process changes drastically the morphology of the microcarrier. Although the substrate morphology is of critical importance in the bioprocesses, accurate data on the 3D morphology of microcarriers in their wet state is hardly available. For most microcarriers, the available information is insufficient to quantify and analyse their morphological variability. At best, some microcarrier size and surface area ranges are given by the manufacturers.

While microscope or SEM-based imaging modalities are commonly used for visual evaluation of cell growth on microcarriers, these techniques are inappropriate for precise full 3D morphological analyses of microcarriers. Indeed, such analyses would require a precise 3D imaging modality that can image the external and internal microstructure of a representative number of microcarriers at the same time, both in their dry and wet state. As it provides a wide field of view to image a large amount of microcarriers at the same time, and since it is a non-destructive imaging technique allowing full 3D imaging of materials, microfocus X-ray computed tomography (microCT) is appropriate for imaging microcarriers in their dry state. However, standard absorption microCT is inappropriate to visualise microcarriers in their wet state, because of their density being close to water. Contrast-enhanced microCT (CE-CT) has been recently reported as an innovative approach for 3D visualisation of soft materials in their wet state^25,26^. CE-CT consists of using high atomic number chemical compounds that bind to specific soft materials in order to increase their X-ray attenuation coefficient, also referred to as contrast-enhancing staining agents (CESAs)^27^.

In this study, we used (i) microCT to image microcarriers in their dry state and (ii) CE-CT to image the microcarriers in their wet state. Together with in-house developed dedicated image processing algorithms, they enabled high throughput, high-resolution visualisation of single microcarriers and their morphological characterization. By imaging microcarriers both in their dry and wet state with the same imaging modality, quantification of the microcarrier swelling and of the attachment surface area were performed. With this study, we provided a publicly available, representative database of 3D morphometrics for the most used microcarriers in adherent cell expansion processes, both in their dry and wet state. This database provides a basis for the optimisation of attachment conditions in microcarrier-based cell cultures, depending on cell size. Additionally, this can provide future studies with more insights in microcarrier morphology-related process variability and enables bioprocess stochastic modelling.

## Results

### 3D visualisation of microcarriers in dry and wet state

**Figure 1:**
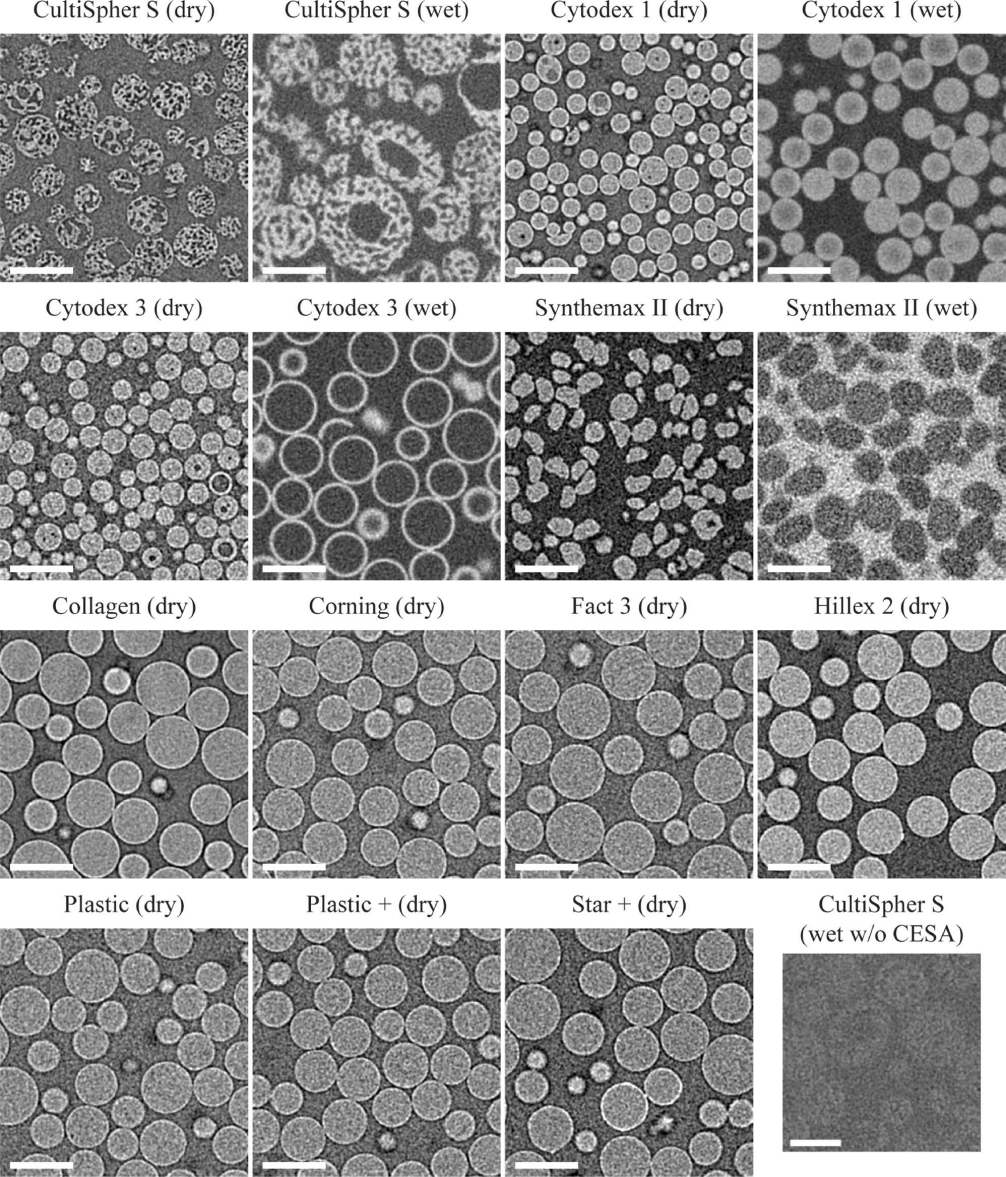
Typical 2D micro-CT and CE-CT cross-sections from all microcarriers types in dry and some in wet state. The bottom right shows a microCT cross-section of CultiSpher S in wet state, without CESA staining. Scale bars = 200 µm.

Figure 1 shows typical 2D cross-sections from all types of microcarriers, for some both in dry and wet state. The cross section of the wet CultiSpher S sample with no contrast agent shows the poor contrast between the microcarriers and PBS and highlights the need to use CESAs for the imaging of such biomaterials in wet state. Swelling due to hydration of the CultiSpher S, Cytodex 1, Cytodex 3 and Synthemax can be observed by comparing the dry and wet state images of these microcarriers. All 2D slices of swelling microcarriers show a general increase in microcarrier size in wet state. Swelling of CultiSpher S microcarriers is also highlighted in Movie 1.

As previously reported, Zr-Kgg POM binds electrostatically to positively charged or collagen containing structures^25^. Figure 1 shows the effective contrast enhancement thanks to the binding of the CESA to the gelatine matrix of CultiSpher S, the positively charged diethylaminoethyl groups of Cytodex 1 and the collagen layer of Cytodex 3. The effective repulsion of Hexabrix by the negative charges of Synthemax II microcarriers is also clear (Fig ure1), making them appear darker than the suspension solution.

### Morphological characterization of the microcarriers

The morphometrics of all microcarrier types, for each microcarrier in the dataset, were gathered in a publicly available Mendeley Data repository accessible here: http://dx.doi.org/10.17632/rf6hsw3f2d.1^52^.

Supplementary Figs. 2 and 3, respectively, show normalized histogram distributions of the equivalent diameters and surface areas for all the microcarrier types, and give an overview of the variability in their morphology.

#### Spherical microcarriers

**Figure 2:**
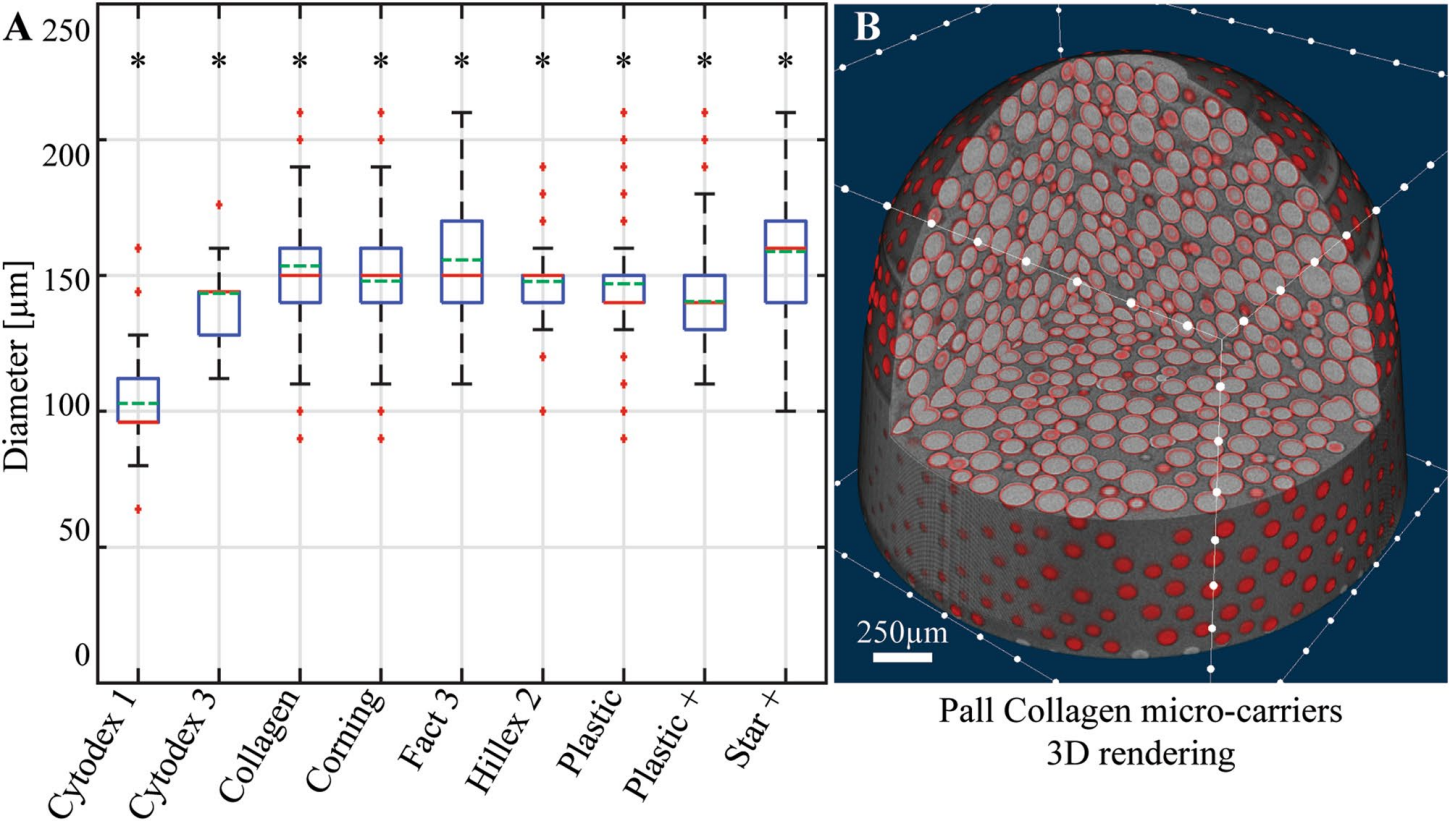
Results of the Hough transform on spherical microcarriers in wet state. (**A**) Calculated spherical microcarrier diameter distribution, shown as box plots. The asterisks indicate the rejection of the hypothesis that the data comes from a standard normal distribution (5% significance level). (**B**) 3D rendering of the Pall Collagen microcarrier dataset (grayscales) overlapped with binary spherical shells (red) calculated from the identified sphere centres and radii detected by the Hough transform.

Figure 2A shows boxplots of the calculated microcarrier diameters for the spherical beads, in wet state when applicable. The radii, surface areas and microcarrier volumes can be found in the repository. None of these morphometrics followed a symmetrical distribution.

Figure 2B and Movie 2 show a 3D rendering of a sample of Pall Collagen microcarriers (grey), along with the fitted spheres from the Hough transform (red). This provides a qualitative assessment of the performance of the algorithm. Good fit was observed between all modelled sphere volumes and their original datasets, for all spherical microcarrier types.

#### Non-spherical microcarriers

**Figure 3:**
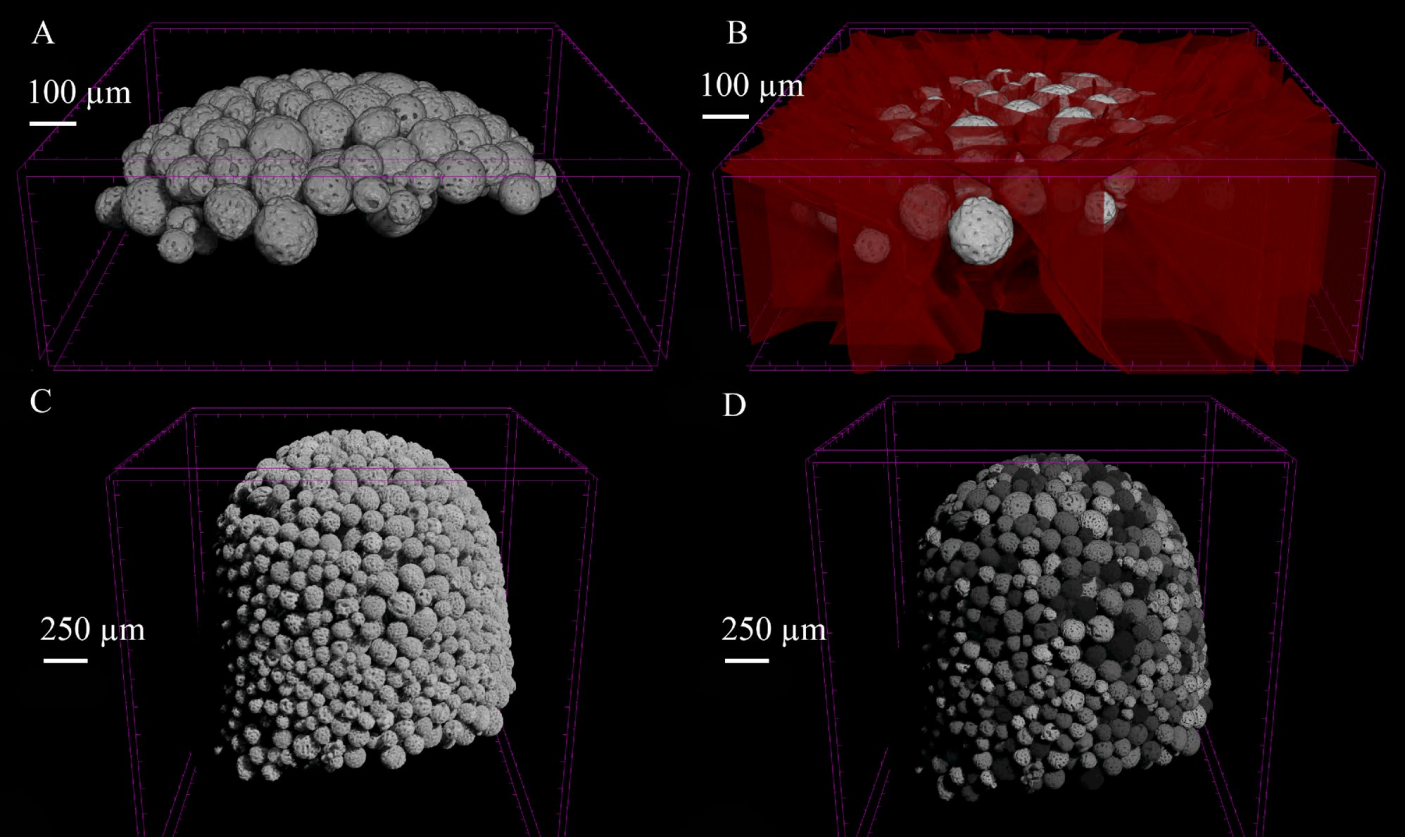
Still of Movie 3. 3D visualisation of the segmentation algorithm results on CultiSpher S microcarriers. (**A**) Binarized validation sample. (**B**) Binarized validation sample (white) overlapped with watershed segmentation contours (red). (**C**) Binarized CultiSpher S sample before segmentation. (**D**) Binarized CultiSpher S sample after segmentation, individual microcarriers were all given a random grayscale value.

1. *Validation of the segmentation algorithm*
  The results of the automatic segmentation algorithm are illustrated in Movie 3 and a still of that Movie is shown on Fig. 3. Figures 3A and 3B show a 3D renderings of validation dataset 1, overlapped with the watershed contour from the segmentation, respectively. Figures 3C and 3D show the CultiSpher S sample in wet state before segmentation and after segmentation, respectively, where all individual microcarriers were assigned a random grayscale value between 30 and 255 to visually assess the quality of the segmentation.
  The manual segmentation of the validation datasets led to the identification and isolation of 249 individual microcarriers, while the automatic segmentation algorithm led to the segmentation of 211 microcarriers, thus showing a 15% underestimation of the number of microcarriers with the automatic segmentation algorithm. The comparison of the distribution of the morphometrics calculated from the two segmentation methods is summarized in Table 2, as validation for the segmentation algorithm. This table shows that the automatic segmentation algorithm leads to similar results than the manual segmentation. However, the pore volume and porosity were significantly different. A significant difference between both segmentation methods was also observed on the Principal axis 1 length, although with a p-value was close to the significance threshold (0.042).
2. *Morphometrics*

**Table 2:** Validation results of the automatic segmentation algorithm against manual segmentation, using distributions of morphometrics (column 1).

	Manual segmentation					T-test		Automatic segmentation				
Morphometric	Avg	Std	Q25	Median	Q75	*α* = *5%*	*p*	Avg	Std	Q25	Median	Q75
Equivalent Diameter [µm]	199.18	72.28	146.83	211.64	243.79		0.144	208.52	63.16	153.73	212.09	245.86
Principal Axis 1 Length [µm]	190.76	73.28	137.09	197.81	232.40	*	0.042	204.00	64.25	157.88	200.69	234.80
Principal Axis 2 Length [µm]	177.76	64.20	129.98	190.05	219.66		0.134	186.21	54.86	138.10	190.77	220.46
Principal Axis 3 Length [µm]	168.16	59.98	125.89	180.48	201.72		0.305	173.64	53.27	133.20	177.02	201.06
Convex surface area [µm^2]	1.41E5	9.04E4	6.71E4	1.40E5	1.88E5		0.253	1.50E5	8.94E4	7.54E4	1.41E5	1.91E5
Bead volume [µm^3]	4.43E6	4.07E6	1.28E6	3.91E6	6.00E6		0.677	4.28E6	3.83E6	1.39E6	3.54E6	5.44E6
Convex hull volume [µm^3]	5.73E6	5.33E6	1.66E6	4.96E6	7.59E6		0.477	6.09E6	5.57E6	1.90E6	5.00E6	7.78E6
Open pore volume [µm^3]	1.24E6	1.29E6	3.69E5	9.52E5	1.58E6	*	1.75E-4	1.79E6	1.80E6	7.39E5	1.40E6	2.14E6
Closed pore volume [µm^3]	5.56E4	1.28E5	1.01E3	1.12E4	4.79E4	*	0.004	2.46E4	9.86E4	3.68E2	3.62E3	1.31E4
Porosity [/]	0.23	0.05	0.20	0.22	0.26	*	8.31E-30	0.30	0.07	0.26	0.29	0.33

For each morphometric (rows), the average value, the standard deviation, the quantile 25%, the median and the quantile 75% are calculated from the manually segmented microcarriers (columns 2–6) and the automatically segmented microcarriers (columns 9–13). Both methods are statistically compared for each resulting morphometric using a Student’s t-test, n = 249 (manual) and n = 211 (automatic) (columns 7–8). * indicates statistical significance (α = 0.05).

**Figure 4:**
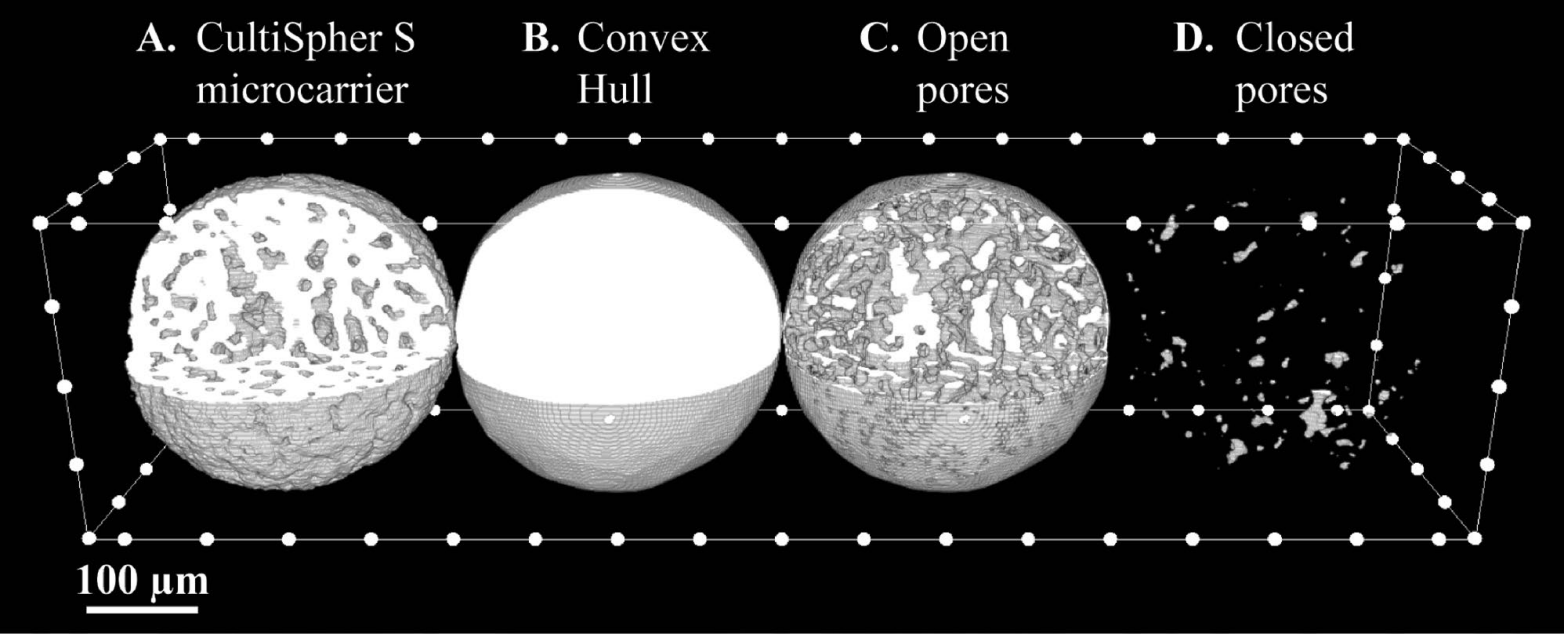
3D visualisation of an individual CultiSpher S. From left to right: the microcarrier (**A**), the calculated convex hull (**B**), open pores (**C**) and closed pores (**D**).

Figure 4 shows the results of the calculation of the convex hull, the open pores and the closed pores on an individual CultiSpher S microcarrier.

**Figure 5:**
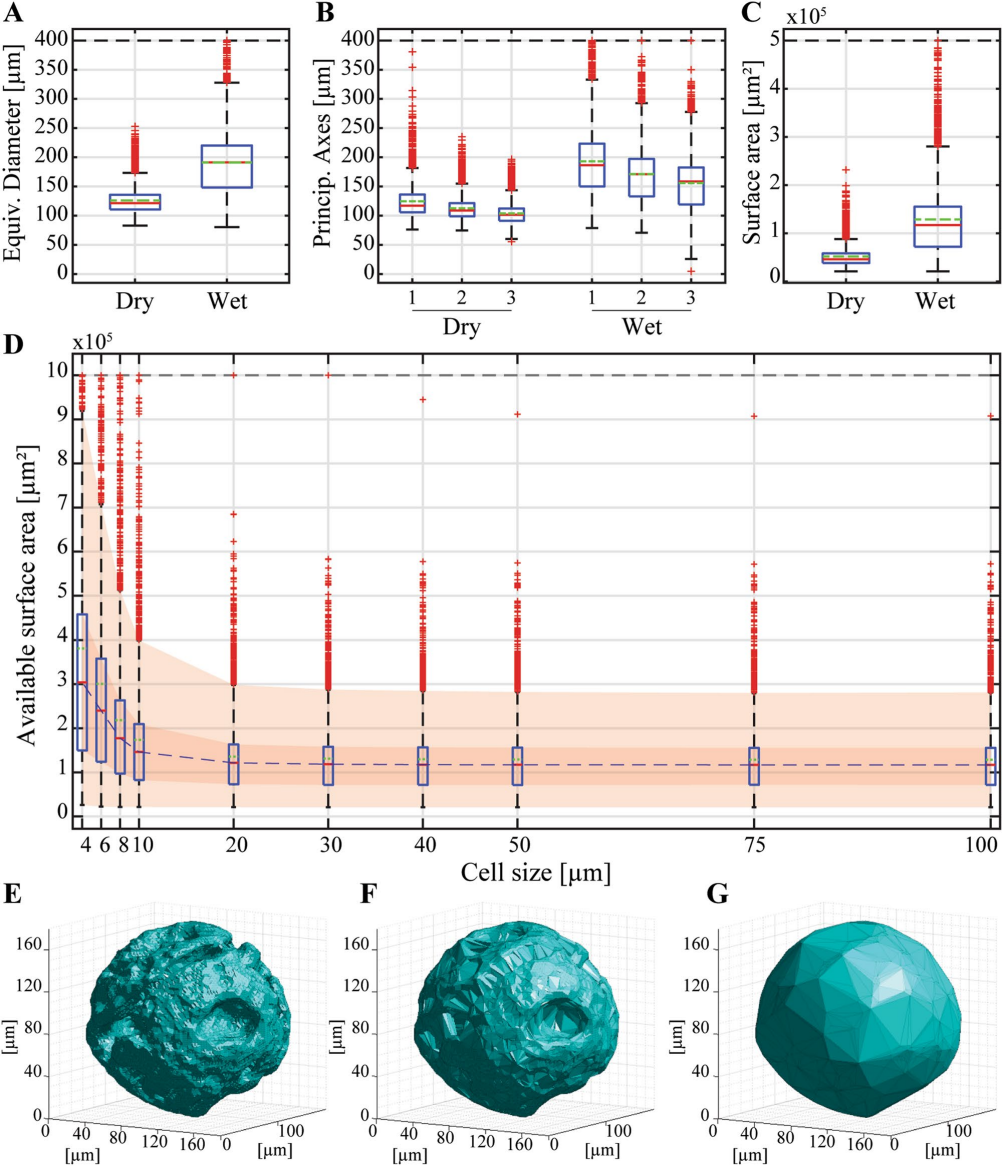
Morphometrics of individual CultiSpher S microcarriers in dry (n = 2820) and wet (n = 2958) state. (**A**) Boxplot distributions of the equivalent diameter. (**B**) Boxplot distributions of the 3 principal axes. The axes are numbered from 1 to 3 in each state. (**C**) Boxplot distributions of the convex surface area. (**D**) Boxplot distributions of the available surface area for cell attachment as a function of the cell size. (**E**–**G**) 3D representations of the calculated alpha shapes for a probing radius (≡ cell size) of (**E**) 4, (**F**) 12 and (**G**) + ∞ µm. The latter corresponds to the convex hull of the microcarrier.

For CultiSpher S, an increase of 51.64% in equivalent diameter (Fig. 5A) and of 147.56% in convex surface area (Fig. 5C) were observed due to swelling. For Synthemax II, swelling led to an increase of 58.77% in equivalent diameter (Suppl. Figure 4A) and of 151.82% in convex surface area (Suppl. Figure 4C). The three principal axes of the microcarriers indicated that the CultiSpher S microcarriers have a generally more spherical shape (Fig. 5B) than the Synthemax II ones (Suppl. Figure 4B), both in dry and wet state. This was confirmed visually (3D renderings of the microcarriers, Fig. 5E–G and Suppl. Figures4E–G). Varying the probing radius of the alpha shapes allowed visualisation of the accessible microcarrier surface area for a given cell size (Fig. 5E–G and Suppl. Figures4E–G). The available surface area was also plotted as a function of the cell size (Fig. 5D and Suppl. Figures4D). For small cell sizes (~ 4 µm), the median available surface area for cell attachment increased by a factor 2.60 for CultiSpher S and 1.08 for Synthemax II, compared to the convex surface area, accessible to bigger cells (≥ 50 µm).

**Figure 6:**
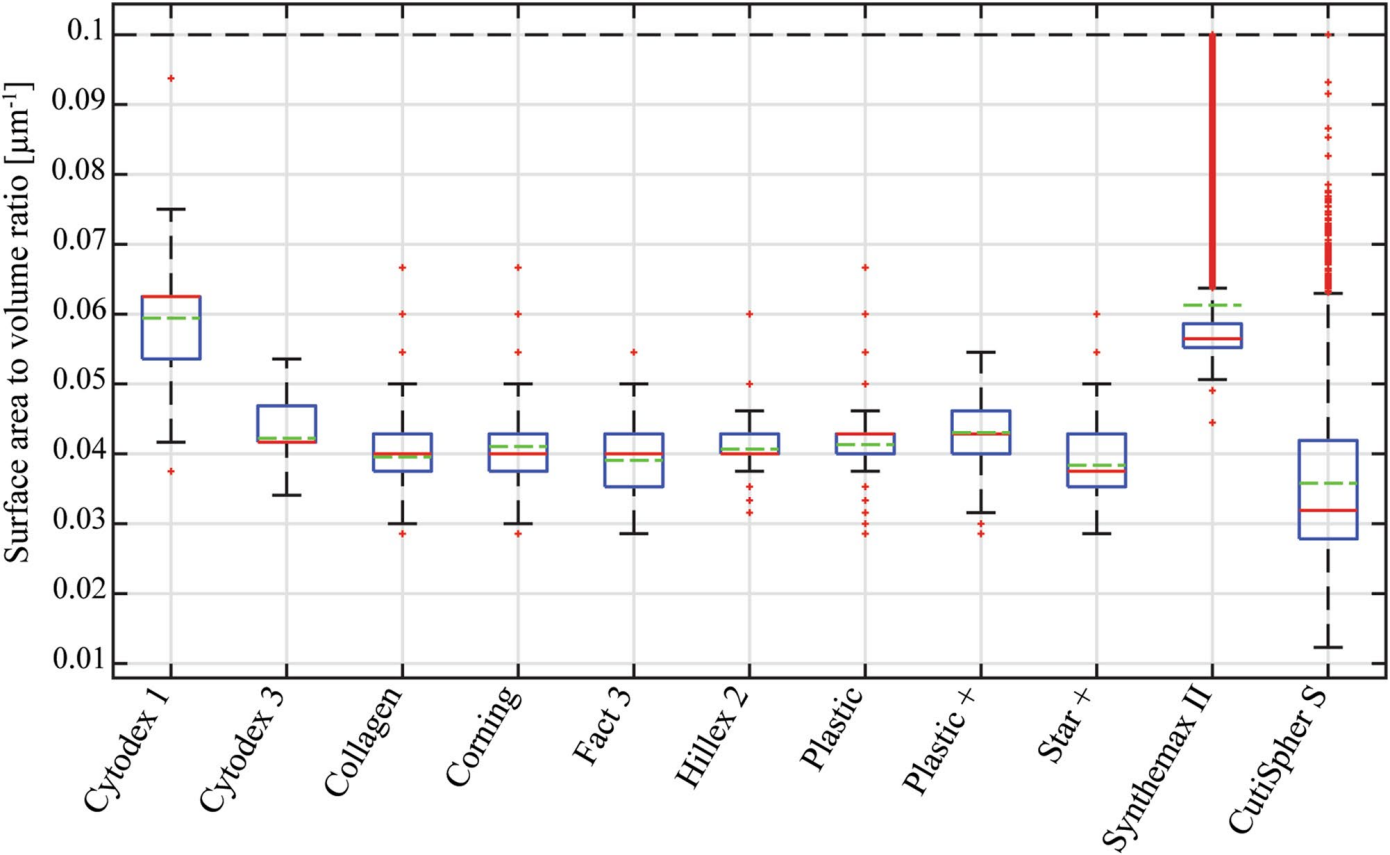
Boxplot distributions of surface area to volume ratio for all microcarrier types, in wet state.

The distribution of the surface area to volume ratio (Fig. 6) shows that the non-spherical microcarriers, Cytodex 1 and Synthemax II, had the highest surface area to volume ratio.

### Comparison to the microcarrier specifications from the manufacturers

Supplementary Table 3 compares the morphometrics calculated in this study (for the swelling microcarriers) to the morphological information specified by the manufacturers (accessible attachment surface area per dry weight and estimated microcarrier size). Similar values to the manufacturers were found for the non-swelling microcarriers. Lower values were found for our calculations for the morphometrics of the swelling microcarriers, especially for the surface area per dry weight and the size of the Cytodex microcarriers.

## Discussion

### 3D visualisation of microcarriers in dry and wet state

This study showed that, by using the proper CESA for each biomaterial, the combined use of microCT and CE-CT is a highly suitable technique to image microcarriers both in their dry and wet state (Fig. 1). The generated datasets provided full 3D imaging of high numbers of microcarriers per sample, which was suitable to analyse the morphometrics from each individual microcarrier. The morphometrics were gathered in a database and their histogram distributions show the large variability in morphological properties of microcarriers in one batch. They form the basis for accurate stochastic modelling of microcarrier-based bioprocesses. To the best of our knowledge, this study is the first report in the field showing full 3D morphometrics of individual microcarriers. For swelling microcarriers, a change in the morphometric distribution was observed after swelling, indicating that microcarriers are affected unequally by the swelling process; this induces even increased variability.

### Validation of the segmentation algorithm

There was good agreement between the morphometrics determined using our in-house developed automatic segmentation algorithm and the ones determined using manual segmentation. However, significant differences were observed for morphometrics related to porosity (porosity, open pore volume, closed pore volume), which can be explained by the sensitivity of those morphometrics to the accuracy of the segmentation algorithm. A small over-segmentation of one microcarrier will introduce high concavity in the morphology of that microcarrier, which will directly influence those parameters. A significant difference between the two methods was also observed for the first principal axis of the microcarrier, which can also be explained by the sensitivity of this parameter to over-segmentation.

The underestimation of the number of microcarriers likely comes from the high number of small microcarriers, as observed on the manually segmented dataset (black arrows on Supplementary Figs. 5 and 6). Such small microcarriers are less easily detected by the automatic segmentation algorithm, and therefore induce a bias in the statistics. Additionally, their presence highlights the morphological heterogeneity of CultiSpher S microcarriers.

In general, the results show that the segmentation algorithm performed well, with limitations regarding porosity and a small over-estimation of the first principal axis. However, manual segmentations are prone to errors and both intra- and inter-operator variations. Thus, further manual segmentations should be performed to increase the accuracy of the ground truth dataset and decrease operator bias.

### Morphometrics of microcarriers

Spherical microcarriers (polystyrene and dextran matrices) showed narrower equivalent diameter and surface area distributions than the non-spherical ones (Suppl. Figures 2 and 3), which indicates a more controlled manufacturing process. Narrow morphometric distributions are more suitable for standard use in bioprocesses, as they provide more homogeneous cell culture conditions. In addition to providing a less homogeneous culture environment, attention should be paid to high numbers of small microcarriers in the distributions (i.e. for CultiSpher S and Synthemax II, Supplementary Fig. 2). Indeed, while these microcarriers increase the total available surface area for attachment, it is likely that the attachment behaviour of cells on these microcarriers with higher surface curvature will be different^53^, which again generally increases the heterogeneity in the process.

The Alpha-Shape method allowed to relate cell size (≡ probing radius) with accessible attachment surface area in the case of porous (CultiSpher S) and concave (Synthemax II) microcarriers. The increase in accessible surface area for cell attachment was more pronounced for CultiSpher S than for Synthemax II, due to their macro-pores. However, the additional attachment surface area provided by these pores can only be significantly accessed by small cells (10 µm and smaller, cf. Fig. 5D). These results suggest that the throat size of the CultiSpher S pores is rather small, and that the concave and porous morphologies of Synthemax II and CultiSpher S microcarriers only play a significant role for small cells. Thus, cell size should be accounted for when calculating the seeding density for bioprocessing.

The differences observed between the morphometrics and the data provided by manufacturers for wet microcarriers may be due to their swelling. Swelling generally broadens the histograms of morphometrics, which makes their comparison to average values less relevant. Additionally, the estimates of number of microcarriers per dry weight is highly sensitive to artefacts in the segmentation algorithm, which might also explain these discrepancies. Further studies should be performed on the swelling behaviour and the quantification of the number of microcarriers per dry weight.

## Conclusions

In this study, we showed that CE-CT could be used as a novel technique to image soft biomaterials, such as microcarriers, in their wet state. Combining with standard microCT allowed to assess the swelling of the microcarriers. Both microCT and CE-CT enabled the precise, full 3D imaging and morphological characterization of different types, and high and representative numbers, of microcarriers. Dedicated 3D image processing and analysis (partly developed in-house) allowed quantification of the morphometrics of individual microcarriers (collected in an online database), and the assessment of the swelling behaviour of some microcarrier types. The representative morphometric distributions of all microcarriers form the basis for optimisation of cell culture conditions and bioprocess modelling, in which accurate variability studies can now be conducted. With this study, we have shown that CE-CT can be recognised as innovative quality control tool for the morphology of microcarriers in wet state, and that it can be used for the optimization of new microcarrier-based bioprocesses. In the future, the development of a cell-specific CESA should be considered, to enable the visualisation of single cells on microcarriers, and understand better their attachment behaviour.

## Methods

### Microcarriers

Several commercially available and commonly reported microcarriers for use in large-scale cell expansion experiments were selected for this study and are shown in Table 1.

### Swelling and staining of the microcarriers

For the CultiSpher S, Cytodex 1 and Cytodex 3 microcarriers, we used a polyoxometalate (POM) as CESA for CE-CT. A 3.5% (35 mg/ml) staining solution of a Zirconium-substituted (Zr-Kgg) POM was prepared in PBS^54^. The microcarriers were incubated in the solution over night to allow them to swell and have the CESA staining them. The staining time was optimized for the microcarriers by evaluating for full diffusion of the CESA within the matrix.

The Synthemax II microcarriers were hydrated in PBS (0.15 mL per milligram of microcarriers) for 10 min, according to the manufacturer’s protocol. Because the observed repulsion of those microcarriers to negatively charged CESAs, such as the POM, we used Hexabrix as a CESA for these microcarriers. Hexabrix contains a negatively charged ioxaglate^55,56^. Hexabrix was added to reach a concentration of 10% (1 ml Hexabrix in 10 ml PBS). This CESA provided a ‘negative’ staining of the microcarriers, i.e. the surrounding liquid was contrasted, but the microcarriers were not, as the CESA did not stain them.

### MicroCT and CE-CT image acquisition

A sample of 6–10 mg was taken for each microcarrier type. All microcarrier samples were first imaged in Eppendorf Tubes in dry state, and then in wet state, for the swelling microcarriers (cf. Table 1). All samples were imaged using a Phoenix NanoTom M (GE Measurement and Control Solutions, Germany) with a voxel size of 2 µm, 1200 images per rotation, in fast scan mode (average of 1 and Skip of 0), with a diamond-coated tungsten target in modus 0, using a collimator and no filter. For the samples in dry state, a voltage of 50 kV, a current of 160 µA, and an exposure time of 750 ms were used. For the CultiSpher S, Cytodex 1 and Cytodex 3 samples in wet state, a voltage of 70 kV, with a 120 µA current and a 500 ms exposure time were used. Finally, for the Corning dissolvable Synthemax II (further referred to as Synthemax II) samples in wet state, a voltage of 60 kV, with a current of 140 µA and an exposure time of 1000 ms were used.

All 3D renderings of CT and CE-CT datasets presented here were generated using the CTvox software v3.1.1 r1191 (64-bit) (https://www.bruker.com/products/microtomography/micro-ct-software/3dsuite.html, Bruker Micro-CT, Kontich, Belgium).

### Image processing

All datasets were first processed in the CTAn software v1.17 (https://www.bruker.com/products/microtomography/micro-ct-software/3dsuite.html, Bruker Micro-CT, Kontich, Belgium) to remove the Eppendorf Tubes from the images by drawing circular regions of interest (ROIs). Then the datasets were processed with Matlab 2017b (MathWorks, MA, USA) as described below. Different protocols were applied for the spherical and non-spherical microcarriers.

#### Image analysis of spherical microcarriers

The datasets of the spherical microcarriers (cf. Table 1—rows 1 to 4) were first resized prior to image analysis, for computational efficiency. Then, a 3D spherical shape recognition algorithm (Hough transform) was implemented, using an algorithm previously developed by Xie et al.^57^, with a minimum and maximum radius, ${R}_{min}$ and ${R}_{max}$ respectively. From the Hough transform, the radius and centre of the spherical microcarriers could be calculated. From the radius of each of the single microcarriers, i.e. ${R}_{i}$, the approximate surface area and volume were calculated as $4\pi {R}_{i}^{2}$ and $\frac{4}{3}\pi {R}_{i}^{3}$, respectively. The minimum and maximum radius were chosen according to information provided by the manufacturer. The radii for all spherical microcarrier datasets can be found in Supplementary Table 1. To visually assess the fit between the results from the Hough transform and the datasets, 3D binary masks were built for each sample, containing binary shells (thickness 10 µm) whose centres and radii were taken from the results of the Hough transform.

#### Image analysis of non-spherical microcarriers

For the non-spherical microcarriers (cf. Table 1 – rows 5 to 11), first a segmentation algorithm was applied on the datasets to isolate single beads. Then, individual microcarriers were analysed and morphometrics were calculated. All analyses were performed in Matlab. The segmentation algorithm consisted of the following steps:

- Step 1 Binarization using the automatic multilevel Otsu’s method^58^ with two thresholds, as the images consisted of two background peaks and one microcarrier peak in the histogram. For the wet Synthemax II microcarriers, the grayscale values between the two thresholds were selected as foreground (microcarrier) because of the negative staining, while for the CultiSpher S microcarriers, the grayscale values above the highest threshold were selected as foreground because of the positive staining (see histogram on Supplementary Figure S1).
- Step 2 From the binary images, all objects (defined as connected components with a 3D connectivity of 18) with a volume smaller than ${V}_{small}$ were removed to filter out noise, using the function *bwareaopen*, creating MASK1.
- Step 3 Using the function *bwdist* on MASK1, the Euclidean distance transform of the background was computed. This distance transform was then binarized using a threshold radius ${R}_{p}$ (referring to half of the pore thickness) to define MASK2, containing all the voxels situated at a distance greater than ${R}_{p}$ in the image, hence disconnecting the pores from the background.
- Step 4 A binary morphological opening of the MASK2 was performed with the function *imopen*, using a spherical structuring element of radius ${R}_{op}$, to disconnect the remaining pores from the background, resulting in MASK3.
- Step 5 By using the function *imclearborder* on MASK3, the remaining pore objects were isolated and were cleared out of the mask by combining it with the inverse of the isolated pore objects, in a Boolean operation AND.
- The resulting binary object was then dilated using a binary morphological dilation with a spherical structuring element of radius ${R}_{p}$, allowing to dilate the edge of the background object back to the microcarrier boundary, creating MASK4.
- Step 6 The inverse of MASK4 is a binarization of touching microcarriers, with closed pores. Thus, a distance transform combined with a watershed segmentation, as described by Fernand Meyer^59^, was used to segment the different microcarriers (function *watershed*). To decrease segmentation artefacts, the distance transform was filtered with a gaussian filter (function *imgaussfilt3* with standard deviation 7).

Intermediate results of this algorithm, applied to CultiSpher S microcarriers in wet state, are shown in Supplementary Fig. 1. The parameter values used for every dataset are listed in Supplementary Table 2. After segmentation, all microcarriers in each dataset were individually analysed in 3D to compute the morphometrics and their distributions per sample.

As the Synthemax II microcarriers are not porous, Steps 3 and 5 were skipped for these datasets. Also, an intermediate closing (with radius ${R}_{cl}$) and removal of small black volumes were added to remove background noise emerging from the negative staining. For Synthemax II, the function *regionprops3* was used to compute the microcarrier volume, equivalent diameter (assuming a sphere of the same volume) and the three principal axis lengths, calculated as the length of the major axes of the ellipsoid that have the same normalized second central moments as the volume of the microcarrier.

For the CultiSpher S datasets, both in dry and wet state, *regionprops3* was first used to compute the convex hull image of the microcarrier. Let ${I}_{i}$ and ${I}_{conv,i}$ respectively be the 3D binary images of microcarrier $i$ (Fig. 4A) and its convex hull (Fig. 4B). By inverting ${I}_{i}$ and clearing all objects connected to the image borders, the closed pores ${I}_{cl,i}$, of the microcarrier can be found (Fig. 4D). From these, the compact microcarrier (filled closed pores) was defined as ${I}_{fill,i}={I}_{i}\bigvee {I}_{cl,i}$ and the open pores of the microcarrier can be calculated as ${I}_{op,i}={I}_{conv,i}\bigwedge \left[\neg {I}_{fill,i}\right]$ (Fig. 4C). Summing up the true voxels of these variables allows the calculation of the microcarrier volume ${V}_{i}$, the convex hull volume ${V}_{conv,i}$, the open pore volume ${V}_{op,i}$ and the closed pore volume ${V}_{cl,i}$. Porosity of an individual microcarrier was calculated as ${P}_{i}=1-\frac{{V}_{i}}{{V}_{conv,i}}$. Using *regionprops3* on ${I}_{conv,i}$ allowed calculation of the equivalent diameter, the three principal axis lengths and the convex surface area.

For calculating the surface area of both Synthemax II and CultiSpher S microcarriers, an alpha-shape method was implemented, as described by Edelsbrunner et al.^60^. Varying the probing radius allowed to relate the available surface area for cell attachment to the cell size. An infinite probing radius returned the convex surface area of the microcarrier, which was confirmed by comparing this technique to the Crofton’s formula^61^ (implemented in *regionprops3*) applied on ${I}_{conv,i}$. Thus, the 3D coordinates of the foreground pixels of ${I}_{i}$ were used as set point input to build the alpha shapes with probing radii ranging between $\left[2 , 3 , 4 , 10 , 15 , 20 , 25 , 38 , 50 , +\infty \right]$ voxels, therefore relating to cell sizes ranging between $\left[4 , 6 , 8 , 20 , 30 , 40 , 50 , 76 , 100 , +\infty \right]$ µm.

To validate our segmentation algorithm, a manual segmentation of all individual microcarriers was performed on two smaller samples of CultiSpher S microcarriers. The individual segmentations were performed using the ROI drawing tool of CTAn (Bruker Micro-CT, Kontich, Belgium), each time saving single microcarrier datasets and removing them from the sample dataset. The automatic segmentation algorithm was also on both datasets. Then, individual morphometrics were calculated on the individual microcarriers, segmented from both techniques, and results were compared. To increase statistical power, both CultiSpher S datasets were combined in each group.

### Surface area per dry weight

From the calculated morphometrics, the attachment surface area per dry weight of microcarrier can be estimated. This parameter is highly relevant to the bioprocess and makes a link between the characterization and the function of the microcarrier. Additionally, it is one the few experimentally determined metrics that can be compared to the morphological information provided by the manufacturers. For all microcarriers that do not swell, this parameter can be calculated as $\frac{\stackrel{\sim }{S}}{\stackrel{\sim }{V}*d}$ where $\stackrel{\sim }{S}$ and $\stackrel{\sim }{V}$ are the median surface area and median volume of the microcarrier dataset (respectively) and $d$ is the density of that microcarrier. For the swelling microcarriers, this parameter was estimated as the median surface area of the microcarriers $\stackrel{\sim }{S}$ multiplied by the number of microcarriers per dry weight (Table 1). However, since this number was not available for the Synthemax II, it was estimated from the ratio of the number of microcarriers imaged in the dry sample, over the sample weight.

### Statistical analyses

To quantify significant differences between morphometrics of CultiSpher S calculated from the two different segmentation methods, a paired t-test was performed in Matlab. To test the null hypotheses of standard normal distribution of morphometrics, a one-sample Kolmogorov–Smirnov test was used. In all test cases, p = 0.05 was considered significant.

The multiple boxplots shown throughout the paper should be interpreted as follows: the green bars indicate the average value, the red bars show the median values, the blue ones show the 25% and 75% quantiles, the vertical dotted black lines show the whiskers (minimum and maximum values without outliers) and the red crosses indicate outliers. Morphometrics were considered outliers if their value was greater than ${q}_{3}+1.5*\left({q}_{3}-{q}_{1}\right)$ or less than ${q}_{1}-1.5*\left({q}_{3}-{q}_{1}\right)$, where ${q}_{1}$ and ${q}_{3}$ are respectively the 25th and 75th percentiles of the morphometric. For clarity of the figures, extreme data limits are used on the boxplots. These are shown as horizontal dotted black lines. Outside these limits, outliers are displayed as clipped at the limit.

## Supplementary information

Supplementary Table 1

Supplementary Table 2

Supplementary Table 3

Supplementary Figure 1

Supplementary Figure 2

Supplementary Figure 3

Supplementary Figure 4

Supplementary Figure 5

Supplementary Movie 1

Supplementary Movie 2

Supplementary Movie 3
