# Supplementary material for: Micro computed tomography with and without contrast enhancement for the characterization of microcarriers in dry and wet state
Source: Sci Rep. 2021 Feb 2;11:2819. doi: 10.1038/s41598-021-81998-8 (PMC7854591; doi:10.1038/s41598-021-81998-8)
Supplement: Supplementary file 1 — Supplementary Table 1 [file 41598_2021_81998_MOESM1_ESM.docx]

# Supplementary Table 1

Legend: Parameter values per microcarrier type for Hough transform algorithm on spherical microcarriers

| **Microcarrier** | **State (dry/wet)** | $\boldsymbol{R}_{\boldsymbol{min}}$ **(µm)** | $\boldsymbol{R}_{\boldsymbol{max}}$ **(µm)** | **Resize factor** |
| --- | --- | --- | --- | --- |
| Collagen | Dry | 40 | 120 | 0.4 |
| Corning | Dry | 40 | 120 | 0.4 |
| Hillex 2 | Dry | 40 | 120 | 0.4 |
| Fact 3 | Dry | 40 | 120 | 0.4 |
| Star + | Dry | 40 | 120 | 0.4 |
| Plastic | Dry | 40 | 120 | 0.4 |
| Plastic + | Dry | 40 | 120 | 0.4 |
| Cytodex 1 | Dry | 12 | 80 | 0.5 |
| Cytodex 3 | Dry | 12 | 80 | 0.5 |
| Cytodex 1 | Wet | 24 | 104 | 0.25 |
| Cytodex 3 | Wet | 48 | 104 | 0.25 |
