# Supplementary material for: Micro computed tomography with and without contrast enhancement for the characterization of microcarriers in dry and wet state
Source: Sci Rep. 2021 Feb 2;11:2819. doi: 10.1038/s41598-021-81998-8 (PMC7854591; doi:10.1038/s41598-021-81998-8)
Supplement: Supplementary file 2 — Supplementary Table 2 [file 41598_2021_81998_MOESM2_ESM.docx]

# Supplementary Table 2

Legend: Parameter values per microcarrier type for non-spherical microcarrier segmentation algorithm

| **Microcarrier** | **State (dry/wet)** | $\boldsymbol{V}_{\boldsymbol{small}}$ **(pixels)** | $\boldsymbol{R}_{\boldsymbol{op}}$ **(pixels)** | $\boldsymbol{R}_{\boldsymbol{p}}$ **(pixels)** | $\boldsymbol{R}_{\boldsymbol{cl}}$ **(pixels)** |
| --- | --- | --- | --- | --- | --- |
| Corning dissolvable Synthemax II | Dry | 2827 | 2 | / | 3 |
| Corning dissolvable Synthemax II | Wet | 6702 | 2 | / | 3 |
| CultiSpher S | Dry | 22619 | 3 | 3 | / |
| CultiSpher S | Wet | 22619 | 5 | 5 | / |
