# Supplementary material for: Micro computed tomography with and without contrast enhancement for the characterization of microcarriers in dry and wet state
Source: Sci Rep. 2021 Feb 2;11:2819. doi: 10.1038/s41598-021-81998-8 (PMC7854591; doi:10.1038/s41598-021-81998-8)
Supplement: Supplementary file 3 — Supplementary Table 3 [file 41598_2021_81998_MOESM3_ESM.docx]

# Supplementary Table 3

Legend: Calculated median values of critical process morphometrics for each microcarrier type. The manufacturers’ microcarrier size is given in column 2 (diameter or equivalent diameter, in µm). The accessible attachment surface area per dry weight (cm²/g dry weight) is given in columns 3 to 5 as a function of the cell size when applicable (CultiSpher S and Synthemax II).

| **Microcarrier** | **Manufacturer’s data** | | **CE-CT quantifications** | |
| --- | --- | --- | --- | --- |
|  | Size | Surface area | Size | Surface area |
| Cytodex 1 | 190 | 4400 | 96.00 | 1244.98 |
| Cytodex 3 | 175 | 2700 | 144.00 | 1954.32 |
| CultiSpher S | 255 | 7500 | 191.28 | 935.80 (cells ≥50µm)  1169.92 (cells ~10µm)  2433.90 (cells ~4µm) |
| Synthemax II | 250 | 5000 | 120.28 | 1161.83 (cells ≥50µm)  1209.71 (cells ~10µm)  1259.00 (cells ~4µm) |
| Corning | 168.5 | 360 | 150.00 | 389.86 |
| Collagen | 168.5 | 360 | 150.00 | 389.86 |
| Fact 3 | 168.5 | 360 | 150.00 | 389.86 |
| Hillex 2 | 180 | 515 | 150.00 | 357.14 |
| Plastic | 168.5 | 360 | 140.00 | 417.71 |
| Plastic + | 168.5 | 360 | 140.00 | 417.71 |
| Star + | 168.5 | 360 | 160.00 | 365.50 |
