# Supplementary figures and images for: Micro computed tomography with and without contrast enhancement for the characterization of microcarriers in dry and wet state
Source: Sci Rep. 2021 Feb 2;11:2819. doi: 10.1038/s41598-021-81998-8 (PMC7854591; doi:10.1038/s41598-021-81998-8)

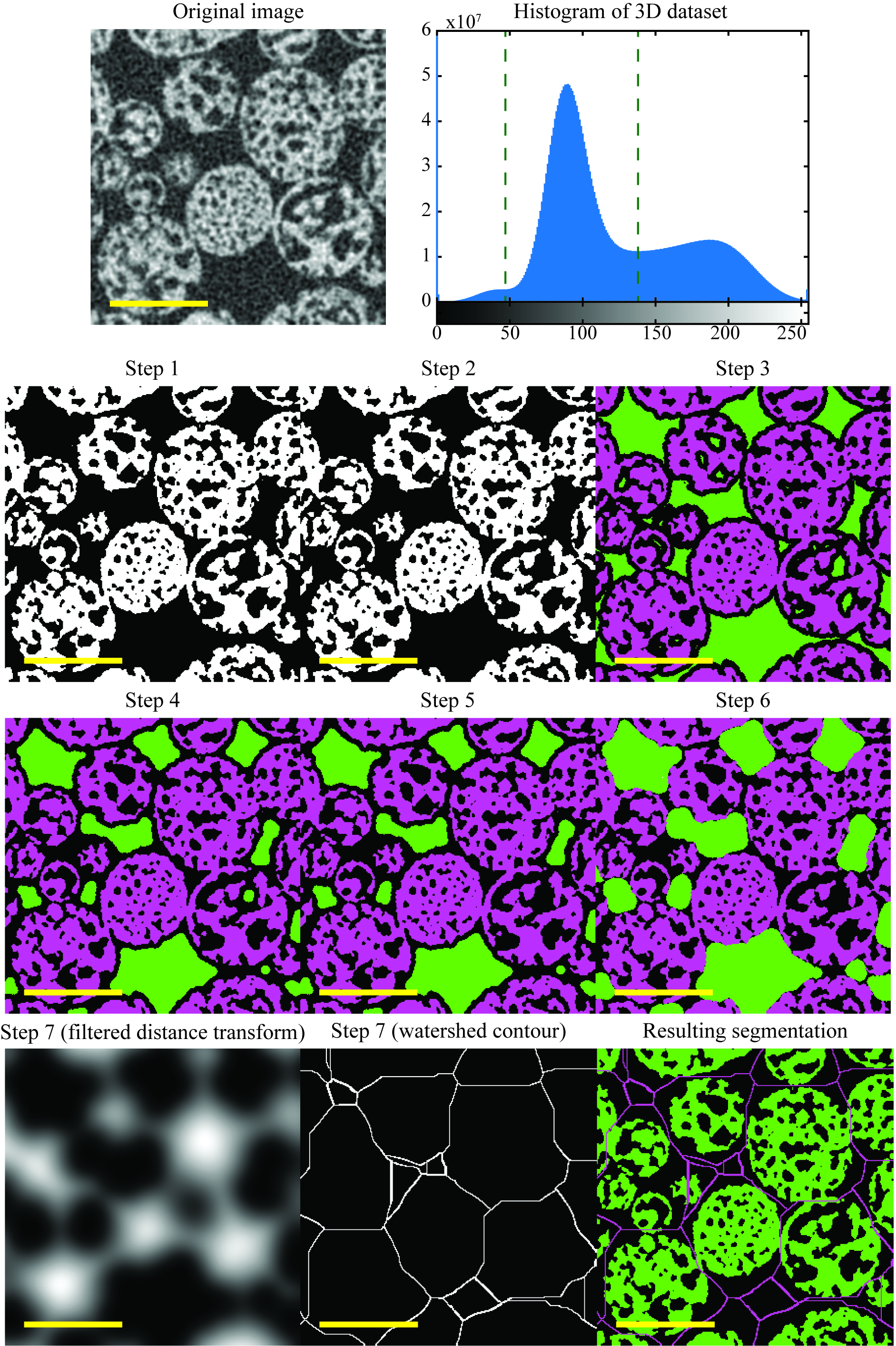

Supplement: Supplementary file 4 — Supplementary Figure 1 [file 41598_2021_81998_MOESM4_ESM.tif]

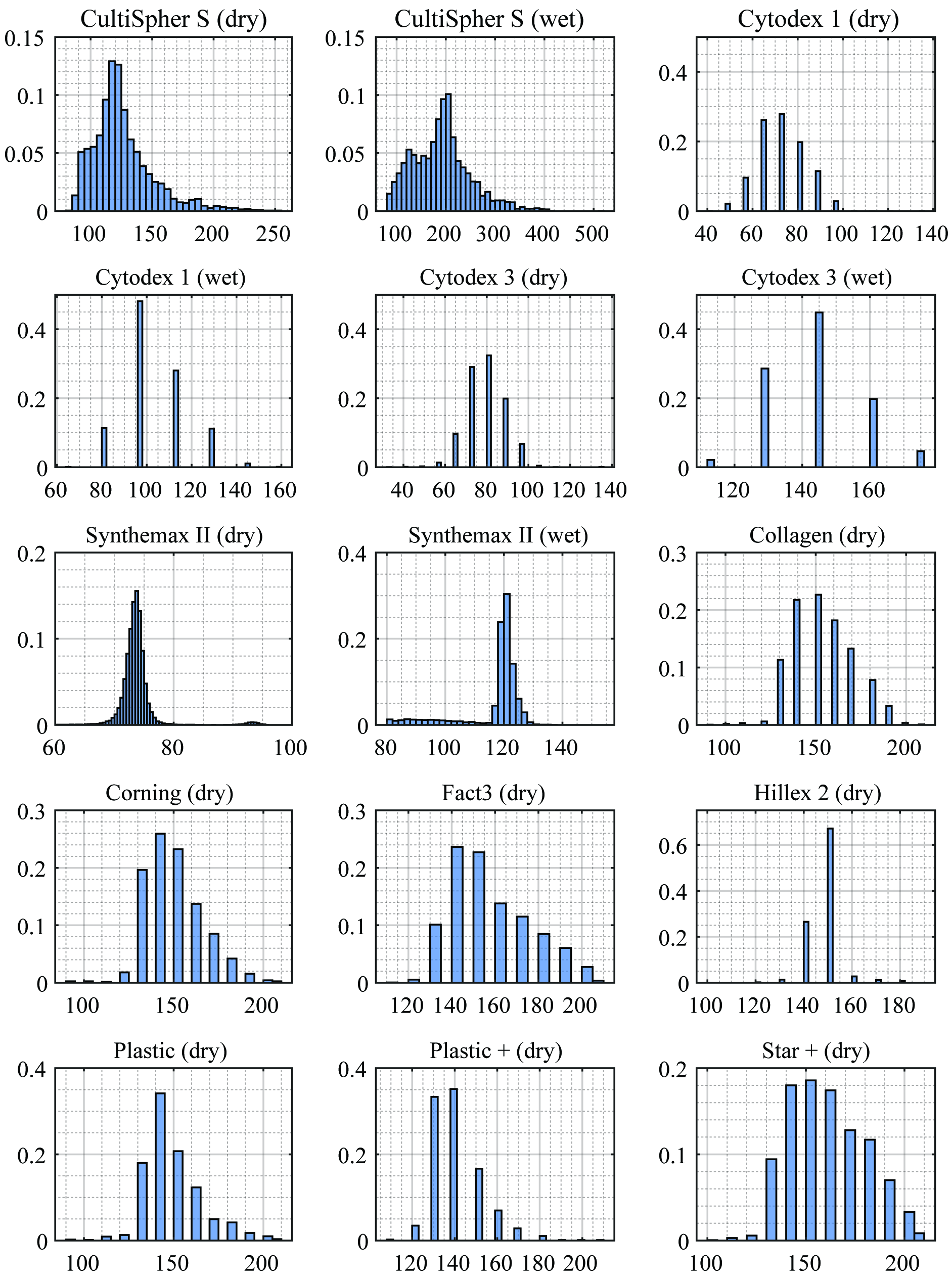

Supplement: Supplementary file 5 — Supplementary Figure 2 [file 41598_2021_81998_MOESM5_ESM.tif]

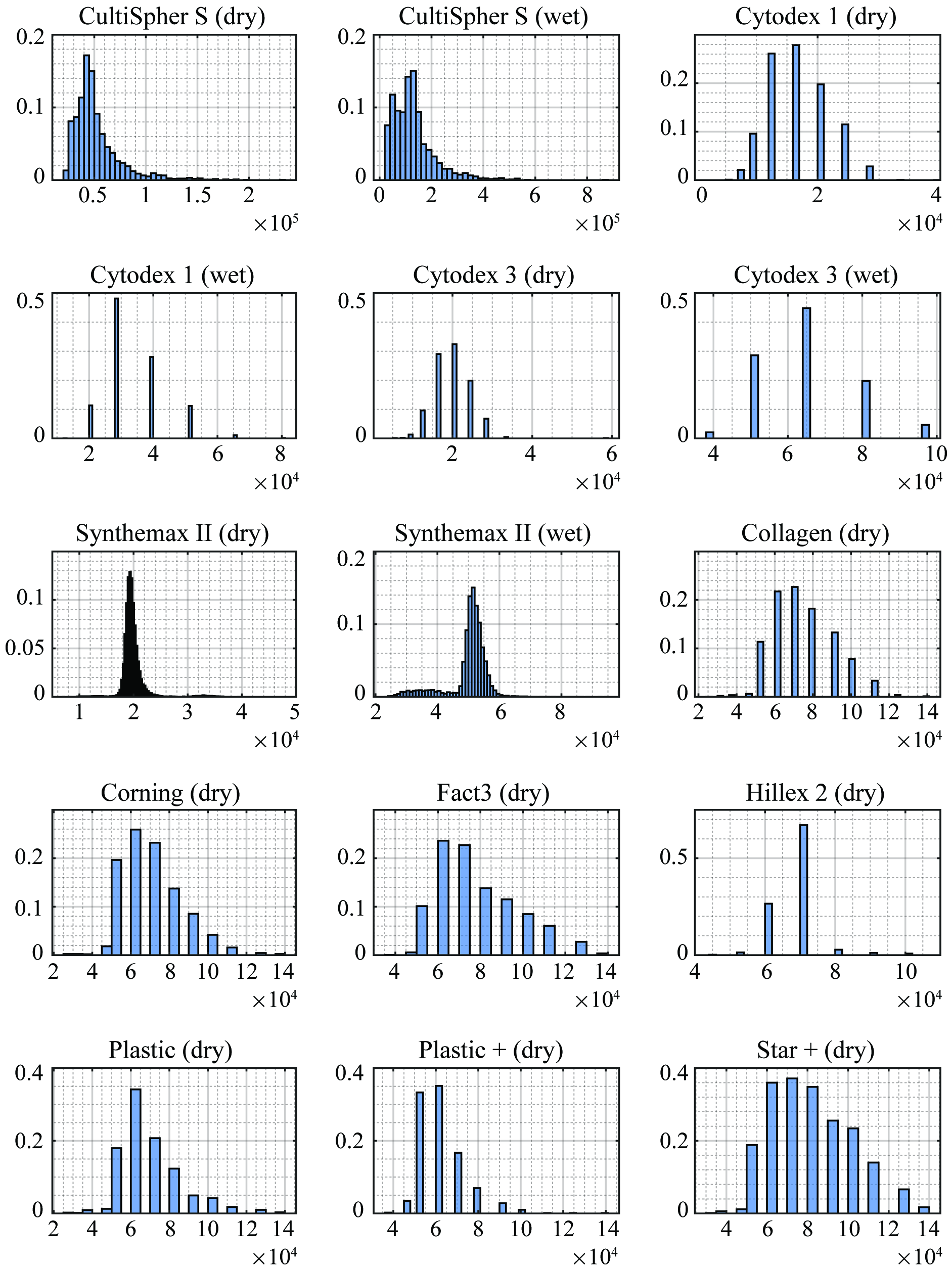

Supplement: Supplementary file 6 — Supplementary Figure 3 [file 41598_2021_81998_MOESM6_ESM.tif]

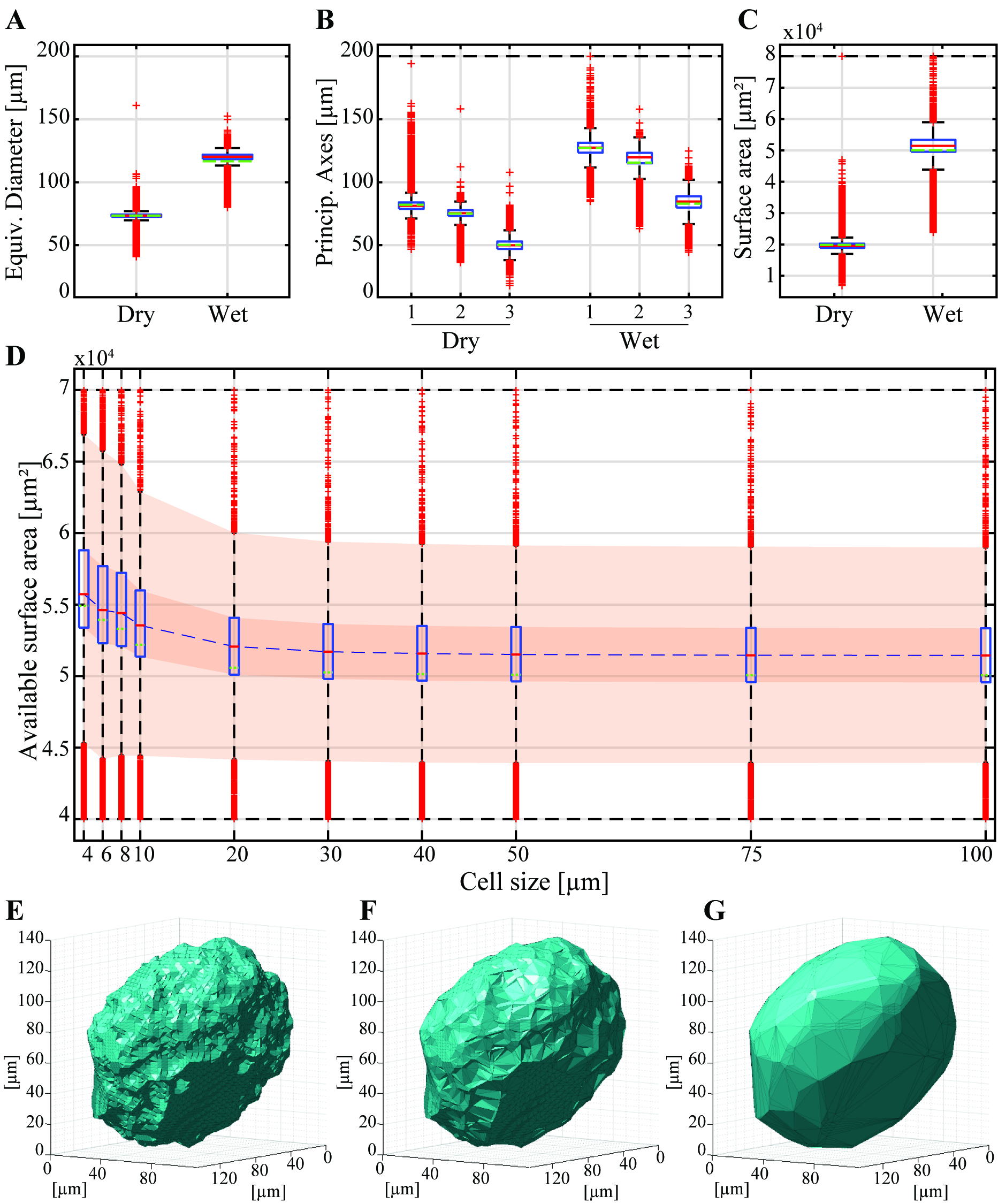

Supplement: Supplementary file 7 — Supplementary Figure 4 [file 41598_2021_81998_MOESM7_ESM.tif]

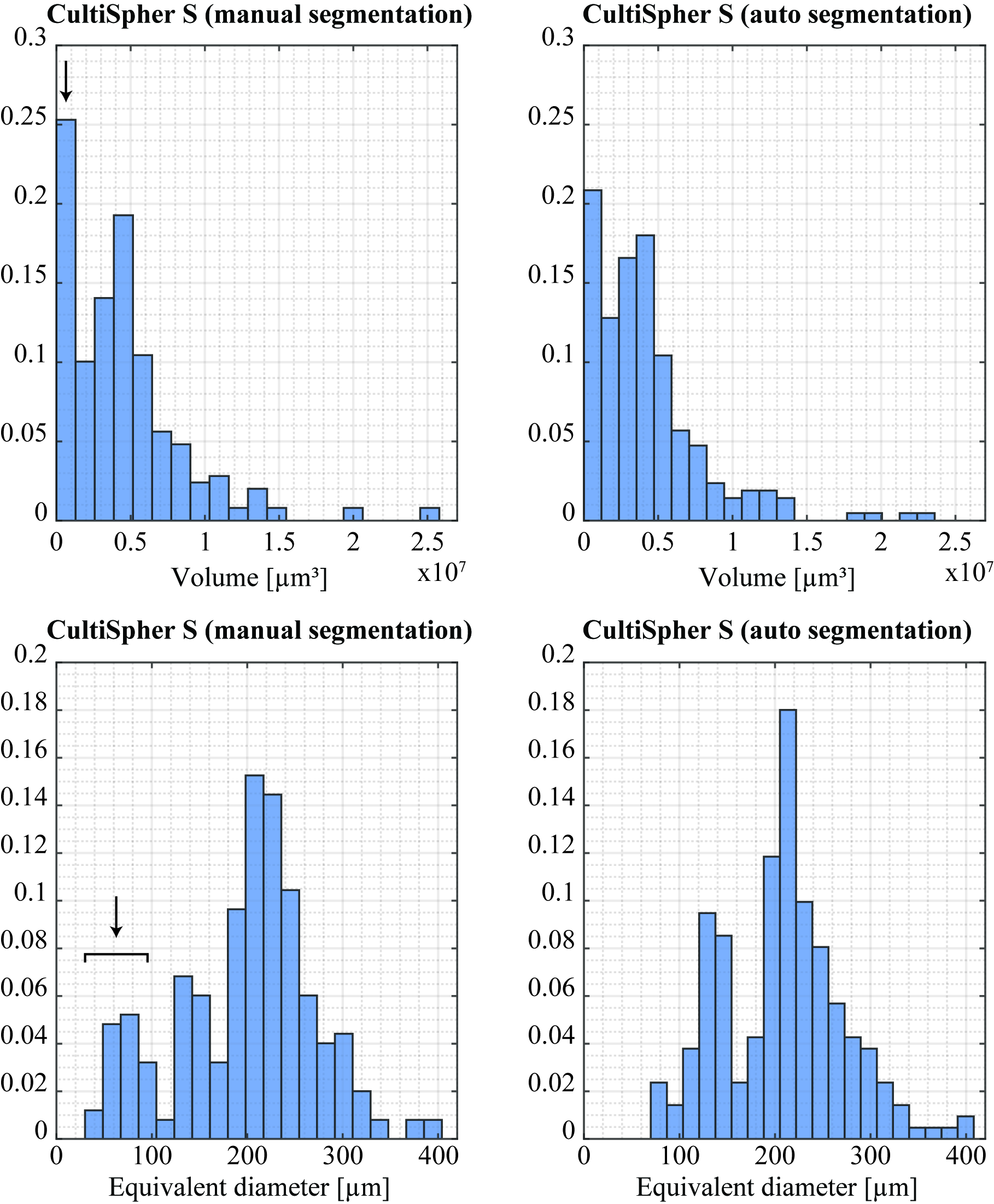

Supplement: Supplementary file 8 — Supplementary Figure 5 [file 41598_2021_81998_MOESM8_ESM.tif]
